# Should Radiation Dose Be Personalized in Patients With Localized NSCLC and Actionable Genetic Alterations? Insight From a Multicenter Real-World Study

**DOI:** 10.1016/j.jtocrr.2024.100774

**Published:** 2024-11-22

**Authors:** Salman Ashraf, Madeha Khan, Nada Naguib, Robert Rulach, Katharine Welsh, Rebecca Carozzi, Ashley Horne, Amelia Payne, Sarah Bowen Jones, Mary Denholm, Georgia Stewart, Ahmed Bedair, Colin R. Lindsay, Stephen Harrow, Corinne Faivre-Finn, Jonathan McAleese, Gerard G. Hanna, Allan Hackshaw, Fiona McDonald, Gerard M. Walls

**Affiliations:** a Department of Clinical Oncology, Cancer Centre Belfast City Hospital, Belfast, United Kingdom; b Department of Thoracic Oncology, Royal Marsden NHS Foundation Trust, London, United Kingdom; c Department of Clinical Oncology, Beatson West of Scotland Cancer Centre, Glasgow, United Kingdom; d Department of Clinical Oncology, The Christie NHS Foundation Trust, Manchester, United Kingdom; e Division of Cancer Sciences, The University of Manchester, Manchester, United Kingdom; f Department of Oncology, Cambridge University Hospitals NHS Foundation Trust, Cambridge, United Kingdom; g Early Cancer Institute, University of Cambridge, Cambridge, United Kingdom; h Department of Clinical Oncology, North West Cancer Centre, Derry, United Kingdom; i Department of Medical Oncology, The Christie NHS Foundation Trust, Manchester, United Kingdom; j Department of Thoracic Oncology, Edinburgh Cancer Centre, Edinburgh, United Kingdom; k Discipline of Radiation Therapy, Trinity Centre for Health Sciences, Trinity College Dublin, Dublin, Ireland; l Cancer Research UK and UCL Cancer Trials Centre, University College London, London, United Kingdom; m Thoracic Radiotherapy Group, The Institute of Cancer Research, London, United Kingdom; n Patrick G. Johnston Centre for Cancer Research, Queen’s University Belfast, Belfast, United Kingdom

**Keywords:** Non-small cell lung cancer, Radiation therapy, Actionable genetic alterations, Mutations, Locoregional control

## Abstract

**Objectives:**

Radiation therapy (RT) is central to the management of unresectable stage I to III NSCLC. However, the impact of actionable genetic driver alterations (AGAs) on locoregional control (LRC) from RT remains uncertain. A retrospective, multicenter real-world study was undertaken to determine if common AGAs impact LRC after RT.

**Methods:**

Patients who received curative-intent RT for NSCLC between 2018 and 2020 at four centers in the United Kingdom and had available molecular testing were included. Locoregional control was compared in a 1:2 ratio between a group of patients with an AGA and a control group without AGAs. Locoregional control was assessed with competing risks analysis and overall survival was analyzed by Cox regression, adjusting for established prognostic clinical factors.

**Results:**

Data was collected for 185 eligible patients: 50 with an AGA and 135 without. Baseline characteristics, including patient demographics, tumor features, and treatment details were evenly distributed between the two groups. LRC was similar in the AGA and non-AGA groups (39% versus 34%, hazard ratio = 1.13, 95% confidence interval: 0.61–1.984, *p* = 0.84). Actionable genetic driver alterations were not associated with LRC according to multivariable regression analysis. Median overall survival was significantly higher in the AGA group (45 mo versus 26 mo, hazard ratio = 0.64, 95% confidence interval: 0.43–0.96, *p* = 0.044), and all these patients received targeted therapies on relapse.

**Conclusion:**

LRC was comparable in the AGA and non-AGA groups suggesting that there is no role for personalization of RT dose solely due to the detection of an AGA. Nevertheless, survival rates were notably higher among patients with AGAs, likely owing to the availability of efficacious targeted therapies on relapse.

## Introduction

The molecular analysis of newly diagnosed advanced NSCLC has led to important improvements in overall survival (OS). Aberrations in specific genes are recognized as actionable genetic driver alterations (AGAs) owing to the availability of targeted therapies, typically using ‘small molecule’ tyrosine kinase inhibitors (TKIs). Despite the variable quality of tumor molecular testing, which depends highly on the properties of the sample obtained, this technology is now widely available and is primarily applied to patients diagnosed with advanced disease.^1^ By contrast, 60% of patients with NSCLC have early or locally advanced disease^2^ and molecular tumor characterization has typically only been performed on relapse, if clinically appropriate and feasible.^2^ Nevertheless, the integration of (neo)adjuvant systemic therapy in clinical pathways on the basis of molecular NSCLC subtypes,^3^^,^^4^ makes prospective analysis for AGAs essential in resectable early and locally advanced NSCLC.

Given that curative-intent local therapy (radiation therapy [RT] or surgery) is not successful for more than 50% of these patients,^5^^,^^6^ there is an imperative to enhance the treatment paradigms in stage I to III disease. The ADAURA trial recently reported that postoperative TKI in *EGFR*-mutant NSCLC significantly improves disease-free survival and OS in patients with stage IB to IIIA disease.^7^ This landmark trial has led to the licensing of adjuvant osimertinib in this patient group. Similarly, in the unresectable setting, osimertinib as an adjuvant therapy after chemo-RT in place of durvalumab in *EGFR*-mutant stage III disease led to a significant improvement in progression-free survival.^8^ Hypothetically, the potential benefits of adding targeted therapies to definitive local therapy may extend to patients with tumors harboring other AGAs such as *ALK*, *RET*, and *ROS1*. It is therefore anticipated that guidelines will likely advocate for molecular testing in non-metastatic NSCLC clinical pathways in the foreseeable future.

While the tumor molecular profile is likely valuable in selecting patients who may benefit from (neo)adjuvant systemic therapy with definitive treatment of NSCLC, it remains unknown whether the local therapy itself should be modified in the presence of an AGA. At present, guidelines do not advocate for RT dose modifications on the basis of histology or other tumor factors, except for hypofractionation in early-stage disease via stereotactic techniques. Nevertheless, there is limited data on RT outcomes in patients with an AGA, and it is not known if radiation dose and fractionation should be personalized according to AGA status in the unresectable NSCLC setting. The suboptimal availability of high-quality diagnostic biopsy tissue presents a challenge in these patients, making molecular testing less commonly available compared to the surgical population, where specimens typically comprise the entire tumor.

Pre-clinical data^9^, ^10^, ^11^ and small clinical case series^12^, ^13^, ^14^ suggest that *EGFR* mutations are associated with radiosensitization, supporting the potential for RT dose de-escalation in this patient population. This could facilitate reductions in RT toxicity that detract from a patient’s survivorship goals, which is highly important in the often-younger AGA-positive population. Nevertheless, AGA status was generally unknown in clinical trials or large retrospective series investigating RT dose questions, and there is a dearth of radiobiology studies in AGAs other than *EGFR*. To address this gap in the literature, this study hereby compared locoregional disease control (LRC) and survival between patients with and without AGAs after primary RT, with or without systemic treatment.

## Materials and Methods

### Study Design

A multicenter, retrospective study aimed to enroll patients with NSCLC who completed chemoRT between January 2018 and December 2020, with the aim of enrolling patients with AGAs and those with qithoutin a 1:2 ratio. Cancer centers in the United Kingdom (UK) were invited to participate via email through the British Thoracic Oncology Group Trainee Network. All patients with non-squamous NSCLC receiving conventionally fractionated or moderately hypofractionated curative-intent thoracic RT, with or without adjuvant durvalumab, were retrospectively identified at each center. Radiation dose-fractionations were converted into biologically effective doses to aid comparison between regimes, using an α/ß ratio of 10 to represent NSCLC. Patients with tissue-based AGA testing were included in the study, regardless of the testing methodology. The next two chronologically consecutive patients treated at local centers with non-AGA tumors were enrolled in the comparator group. Clinical notes were interrogated for patient and tumor characteristics, RT, and systemic treatment details at the time of initial treatment and at the time of relapse. All study details are reported in accordance with the Strengthening the Reporting of Observational Studies in Epidemiology guidelines.^15^

### Molecular Analysis

Single gene assays, immunohistochemistry, commercial and institutional gene panels, and any other validated local method of AGA testing employed in routine clinical care were permissible for inclusion. Cases with testing for aberrations in any one of *EGFR*, *ALK*, *RET*, *ROS1*, *HER2*, *NTRK*, *MET*, *NRG1,* and *KEAP1* were eligible. Patients with mutations in *KRAS* were not considered AGA cases because of the lack of comprehensive testing available and because these mutations were not technically actionable for most of the follow-up period in the UK, primarily because of limited drug access. Although AGAs are biologically distinct, this study included all driver aberrations as they share key clinical characteristics including early distant dissemination, sensitivity to targeted therapies, and associations with light smoking history.

### Clinical Outcomes

Routine disease response assessments on the basis of local standard-of-care imaging and clinical evaluation were recorded for analysis of local and distant disease control. Local relapse was defined as any relapse within the planning target volume, as determined by the treating team, with input from a thoracic radiologist if required. ‘Out-of-field’ relapse within the same lobe was considered a regional failure. Duration of LRC was defined as the period between the first day of RT until the date of confirmed disease progression/relapse at any sites, on the basis of imaging, or tissue biopsy where performed. Survival time was defined as the interval between the start date of RT and the date of death. Relapse-free survival was defined as the time to disease relapse at any location, or death from any cause.

### Statistical Analysis

Descriptive statistics were used to summarize baseline characteristics. Unpaired *t* tests and Fisher’s exact tests were used to compare continuous and categorical baseline characteristics respectively. Median follow-up was calculated using the reverse Kaplan-Meier method. The cumulative incidence of locoregional disease relapse accounting for the competing risk of death was calculated (Gray test), and multivariable analysis to adjust for relevant clinical covariables was performed using Fine-Gray regression. Factors included in these multivariable analyses were selected a priori on the basis of clinical relevance. Kaplan-Meier analysis (log-rank test) and Cox regression were performed to assess OS trends by AGA status, including key prognostic clinical characteristics. Relapse-free survival was calculated using the Kaplan-Meier method. Time-to-event analyses were planned for all patients with AGAs pooled, compared with patients in the non-AGA group. For patients with missing data in modeled factors, the mean was used. Kaplan-Meier and Fine-Gray univariate regression analyses were performed to assess the effect of durvalumab on LRC and OS in patients with stage III disease with an AGA specifically.

### Ethics and Governance

This study was limited to retrospective medical case note review at each center followed by central collation and analysis of de-identified data. Approval for this service evaluation was granted by the Belfast Health and Social Care Trust as the lead center (Reference Number: 6764), and other centers also obtained approval from their local Caldicott Guardians before participation. Informed consent was not required for this retrospective analysis of anonymized patient data.

## Results

Four UK centers enrolled 185 eligible patients, comprising 50 patients with an AGA and 135 patients without. The following AGAs were detected: *EGFR* (34, 68%), *ALK* rearrangement (11, 22%), *MET* (2, 4%), *ROS1* (2, 4%), and *HER2* (1, 2%). The result of molecular testing from diagnostic tissue was available before treatment completion in most cases (170, 92%). The mean age of the cohort was 69.9 years (range: 34–92), the mean performance status was 1 (range: 0–3), 79% of patients had stage III disease and AGAs were more common in female individuals (60% versus 40%) (Table 1). Patient, staging, and tumor characteristics were equally distributed between the groups, except the AGA group had better fitness (*p* = 0.027) and the control group had more magnetic resonance imaging brain staging (*p* = 0.018). Smoking was more common in the control group, both for the current (27% versus 14%) and previous (61% versus 54%) statuses respectively (*p* = 0.001).

**Table 1:** Baseline Patient, Tumor, and Treatment Characteristics

Characteristics	AGA	Non-AGA	*p* Value
No. of patients	50	135	-
Median age (range)	71.5 (34–92)	70.0 (42–90)	0.80
Sex			0.33
Female (%)	30 (60)	71 (53)	
Male (%)	20 (40)	64 (47)	
ECOG PS			0.027
0	16 (32)	23 (17)	
1	27 (54)	74 (55)	
2	5 (10)	28 (21)	
3	0	7 (5)	
Unknown	2 (4)	3 (2)	
Median CCI (range)	5 (2–7)	5 (1–9)	0.51
Smoking status (%)			0.001
Previous	27 (54)	82 (61)	
Current	7 (14)	36 (27)	
Never	8 (16)	3 (2)	
Unknown	8 (16)	14 (10)	
Subtype (%)			0.19
Adenocarcinoma	49 (98)	119 (88)	
Large cell	0	3 (2)	
Adenosquamous	0	5 (4)	
Other	1 (2)	8 (6)	
PD-L1 range (%)			0.26
<1%	14 (28)	36 (27)	
1%–49%	15 (30)	21 (16)	
≥50%	9 (18)	27 (20)	
Unknown	12 (24)	51 (38)	
T-Stage (%)			0.73
0/X	3 (6)	9 (7)	
1	10 (20)	32 (24)	
2	15 (30)	27 (20)	
3	9 (18)	27 (20)	
4	12 (24)	38 (28)	
Unknown	1 (2)	2 (1)	
N-Stage (%)			0.066
0	11 (22)	24 (18)	
1	2 (4)	24 (18)	
2	28 (56)	60 (44)	
3	8 (16)	27 (20)	
Unknown	1 (2)	0	
Overall TNM Staging (%)			0.41
I	4 (8)	7 (5)	
IIA	3 (6)	5 (4)	
IIB	2 (4)	17 (13)	
IIIA	18 (36)	47 (35)	
IIIB	18 (36)	49 (36)	
IIIC	4 (8)	10 (7)	
Unknown	1 (2)	0 (0)	
PET-CT (%)	50 (100)	134 (99)	>0.99
EBUS (%)	29 (58)	70 (52)	0.51
Brain staging (%)			0.018
MRI	7 (14)	39 (29)	
CT	25 (50)	39 (29)	
None	18 (36)	57 (42)	
Mutation (%)			-
*EGFR*	34 (68)	0	
*ALK*	11 (22)	0	
*MET*	2 (4)	0	
*ROS1*	2 (4)	0	
*HER2*	1 (2)	0	
*KRAS*	0	18 (13)	
Radiation therapy planning (%)			0.90
3D-CRT	0	3 (2)	
IMRT	4 (8)	11 (8)	
VMAT	46 (92)	121 (90)	
Median BED (Gy) (range)	70.1 (62.1–79.2)	70.1 (62.1–84)	0.47
Chemotherapy^a^			0.36
None	24 (48)	73 (54)	
Neoadjuvant	14 (28)	42 (31)	
Concurrent	12 (24)	20 (15)	
Adjuvant durvalumab^a^ (%)	9 (17)	28 (21)	0.68
Cycles of durvalumab^a^ (range)	17 (11–26)	12 (1–26)^b^	0.11
Completed 1-year durvalumab^a^ (%)	1 (11)	5 (18)	>0.99

3D-CRT, three-dimensional conformal radiation therapy; AGA, altered genomic aberration; BED, biologically effective dose; CCI, Charlson Comorbidity Index; EBUS, endobronchial ultrasound-guided biopsy; ECOG PS, Eastern Cooperative Oncology Group performance status; IMRT, intensity-modulated radiation therapy; MRI, magnetic resonance imaging; PD-L1, programmed cell death ligand 1; PET-CT, positron emission tomography–computed tomography; TNM, tumor, nodal and metastatic staging status version 8; VMAT, volumetric modulated arc therapy.

a Only stage III cases were eligible.

b Unknown for two patients.

Treatments were also equally distributed between the two groups (Table 1). The most common RT dose-fractionations delivered were 55 Gy in 20 fractions (28, 56% AGA; 87, 47% non-AGA) and 64 Gy in 32 fractions (12, 24% AGA; 23, 12% non-AGA). Approximately half of the patients received chemotherapy (26, 52% AGA; 62, 34% non-AGA). Similar proportions of stage III cases in each group received durvalumab (9, 17% AGA; 28, 21% non-AGA) although patients with an AGA received a greater number of cycles of durvalumab than those without an AGA (mean: 17 versus 12 cycles). Similar proportions completed one year of durvalumab treatment (11% versus 18%, *p* > 0.99). One patient with an *EGFR* mutation received neoadjuvant osimertinib as bridging therapy before concurrent chemo-RT while completing treatment for a non-lung synchronous primary cancer. Nevertheless, no other patients received neoadjuvant therapy owing to frailty or other reasons.

**Figure 1:**
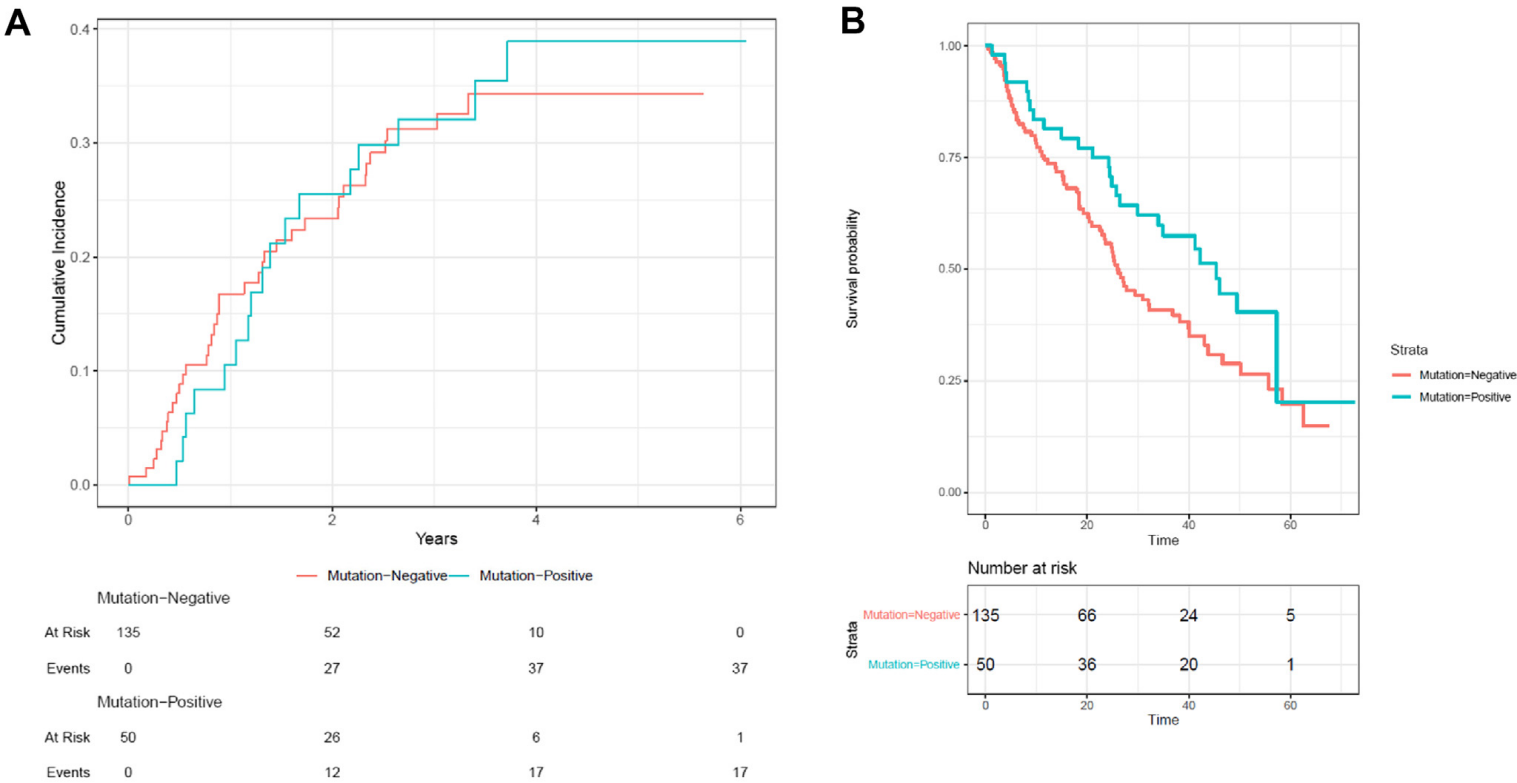
Locoregional relapse *(A)* and overall survival *(B)* for patients with (blue) and without (red) AGAs after curative-intent chemoradiation for NSCLC. AGA, altered genomic aberration.

The median follow-up was 41.1 months (95% confidence interval [CI]: 38.8–48.4) and relapse was recorded in 112 patients (61%), including 54 patients (30%) with locoregional relapse, 84 patients (45%) with metastatic relapse and 52 patients (29%) that had both. Locoregional relapse occurred within six months for one of the 17 (6%) patients with an AGA compared with 10 of 37 patients (27%) without an AGA. The cumulative incidence of locoregional relapse over the entire available follow-up was similar between patients of the AGA and non-AGA groups (38.9% [95% CI: 23.8–53.8] versus 34.3% [95% CI: 25.2–43.7]), after adjusting for the competing risk of death. The difference between the two groups was not statistically significant (hazard ratio [HR] = 1.06, 95% CI: 0.61–1.84, *p* = 0.84) (Fig. 1*A*). AGAs were not associated with the risk of locoregional relapse after adjusting for relevant clinical covariables (HR = 0.98, 95% CI: 0.53–1.83, *p* = 0.95) (Table 2).

**Table 2:** Fine and Gray Multivariable Analysis for Locoregional Relapse

Characteristics	Number of Patients	Number of Events	Hazard Ratio, (95% CI)	*p* Value
Age, increase of 1 y	185	54	1.02 (0.98–1.06)	0.30
Sex				
Male	84	26	Reference	NA
Female	101	28	0.95 (0.54–1.69)	0.86
ECOG PS^a^				
0	39	16	Reference	NA
1	106	27	0.65 (0.34–1.25)	0.20
2	33	9	0.69 (0.27–1.78)	0.44
3	7	2	1.28 (0.19 – 8.82)	0.80
Stage^b^				
1	11	2	Reference	NA
2	27	9	1.67 (0.25–11.44)	0.60
3	147	43	1.36 (0.20–9.01)	0.75
AGA vs. non-AGA	185	54	0.98 (0.53–1.83)	0.95
Radiation therapy BED, increase of 1 Gy	185	54	1.03 (0.95–1.13)	0.48
Chemotherapy				
None	97	26	Reference	NA
Neoadjuvant	56	20	1.19 (0.51–2.79)	0.69
Concurrent	32	8	1.00 (0.31–3.22)	1.00

AGA, altered genomic aberration; BED, biologically effective dose; CI, confidence interval; ECOG PS, Eastern Cooperative Oncology Group performance status; NA, not applicable.

a The mean ECOG PS of 1 was assigned to the five patients where this was missing.

b The mean stage of III was assigned to the one patient where this was missing.

Across the whole cohort, at the time of progression, 46 out of 114 patients (40%) received palliative systemic therapy, 18 out of these 46 patients (39%) for metastatic relapse only, 11 out of 46 patients (24%) for locoregional relapse only, and 17 out of 46 patients (37%) for both locoregional and distant relapse. Of those patients treated with systemic therapy on relapse, the first-line regime was TKIs (20/25, 80%), while five (20%) had chemotherapy, immunotherapy, or a combination of these (Table 3). Cytotoxic chemotherapy (9/20, 45%) was the first-line systemic therapy for patients without an AGA, while 11 (55%) had chemo-immunotherapy. Palliative RT was used for 5/45 patients (11%) and 11/70 patients (16%) with and without AGA, respectively. Radical re-irradiation for local relapse was used for a single patient in the non-AGA group.

**Table 3:** Patterns of Relapse and Subsequent Treatments Administered

Characteristics	AGA	No AGA
Total number of patients	50	135
Relapse (%)		
None	14 (28)	65 (48)
Locoregional	18 (36)	37 (27)
Oligometastatic	11 (22)	19 (14)
Polymetastatic	15 (30)	39 (29)
Any relapse	37 (74)	70 (52)
Repeat definitive radiation therapy (%)	0	1 (1)
Palliative radiation therapy (%)	5 (11)	11 (16)
1L SACT Type^a^ (%)		
Chemotherapy	4 (16)	9 (45)
Immunotherapy	1 (4)	6 (30)
Chemo-immunotherapy	1 (4)	5 (25)
Tyrosine kinase inhibitor	20 (80)	0
2L SACT Type^a^ (%)		
None	13 (52)	12 (71)
Chemotherapy	3 (12)	3 (18)
Immunotherapy	0	2 (12)
Chemo-immunotherapy	1 (4)	0
Tyrosine kinase inhibitor	6 (24)	0

1L, first-line; 2L, second-line; AGA, altered genomic aberration; SACT, systemic anti-cancer therapy.

a Denominator of total patients with relapsed and received first-line palliative systemic therapy was 25 for patients of the AGA group and 17 for patients of the non-AGA group.

During study follow-up, 103 patients (56%) died. The median survival for patients with AGA was greater than those without (45.4 mo [95% CI: 30.0–not reached] versus 25.9 mo, [95% CI: 22.9–38.2]; HR = 0.64, 95% CI: 0.43–0.96, *p* = 0.044) (Fig. 1*B*). On multivariable analysis, lower age and presence of AGA was associated with statistically significant improvements in survival (Table 4). Relapse-free survival was 26.1 months in AGA versus 24.7 months in non-AGA cases (HR = 0.88, 95% CI: 0.59–1.31, *p* = 0.54).

**Table 4:** Cox Multivariable Regression Analysis for Overall Survival

Characteristics	Number of Patients	Number of Events	Hazard Ratio	*p* Value
Age, increase of 1 y	185	103	1.03 (1.01–1.06)	0.015
Sex				
Male	84	50	reference	NA
Female	101	53	0.84 (0.55–1.26)	0.40
ECOG PS^a^				
0	39	23	reference	NA
1	106	55	0.74 (0.42–1.32)	0.31
2	33	21	0.92 (0.45–1.89)	0.82
3	7	4	1.78 (0.54 – 5.87)	0.35
Stage^b^				
1	11	2	reference	NA
2	27	14	2.31 (0.50–10.56)	0.28
3	147	87	3.98 (0.90–17.57)	0.07
AGA vs. non-AGA	185	103	0.59 (0.36–0.95)	0.029
Radiation Therapy BED, increase of 1 Gy	185	103	0.98 (0.92–1.04)	0.51
Chemotherapy				
None	97	58	reference	NA
Neoadjuvant	56	28	0.74 (0.36–1.50)	0.40
Concurrent	32	17	0.96 (0.50–1.85)	0.90

AGA, altered genomic aberration; BED, biologically effective dose; ECOG PS, Eastern Cooperative Oncology Group performance status; NA, not applicable.

a The mean ECOG PS of 1 was assigned to the five patients where this was missing.

b The mean stage of 3 was assigned to the one patient where this was missing.

**Figure 2:**
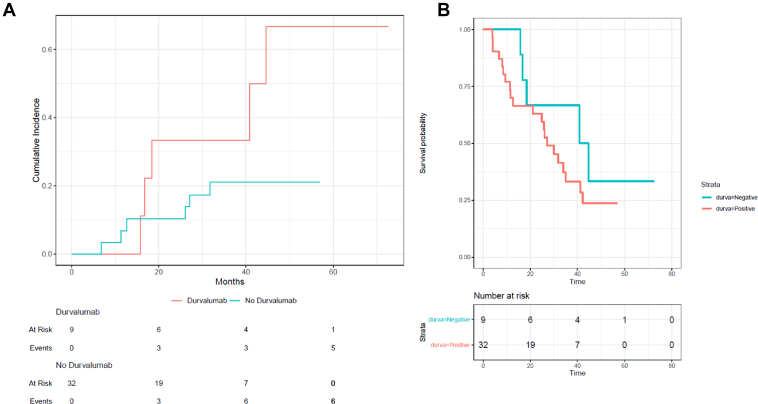
Locoregional relapse *(A)* and overall survival *(B)* in patients with stage III NSCLC and an AGA, comparing those who received adjuvant durvalumab (red) after chemoradiation and those who did not (blue). AGA, altered genomic aberration.

Among 41 patients with AGA-positive stage III disease, treatment with adjuvant durvalumab (n = 9) was not associated with decreased locoregional relapse (HR = 3.03, 95% CI: 0.99–9.09, *p* = 0.053; Fig. 2*A*) or OS (HR = 3.13, 95% CI: 1.27–7.69, *p* = 0.049; Fig. 2*B*) compared with those patients who did not receive it.

## Discussion

Genomic tumor analysis has been widely implemented in advanced NSCLC, enabling clinicians to personalize systemic therapy in patients with an AGA. On the basis of the results of recent studies demonstrating the benefit of osimertinib after definitive surgery with or without adjuvant chemotherapy^7^ or chemo-RT in place of durvalumab^8^ in patients with *EGFR*-mutant disease, testing for AGAs should be performed in stage I to III NSCLC. Another argument in favor of reflex AGA testing is the restriction of licensed preoperative chemo-immunotherapy to those patients without an AGA.^5^ Whether technical aspects of RT should also be tailored according to AGA status has not been explored to date,^16^ primarily owing to the historical lack of molecular testing in this group.

Chemo–radiation therapy predominantly achieves LRC in locally advanced NSCLC; where it does not achieve a cure, it is paramount for managing symptoms and extending survival. As there are limited treatment options available for relapse, radiation dose optimization may be beneficial for this patient population. Equally important, because RT-related toxicity can impair survivorship and survival, and considering that this population is typically slightly younger, fitter, and less burdened by comorbidities compared to non-AGA cases, minimizing radiation exposure of normal thoracic organs is particularly relevant.^17^^,^^18^ Understanding the impact of AGAs on RT response is therefore crucial, to ascertain if RT factors should be individualized for patients with an AGA.

In this multicenter real-world cohort, the locoregional failure rate was not different between patients with or without an AGA, after accounting for other well-established prognostic factors. Nevertheless, OS was better in patients with an AGA compared to those without, likely attributable to the treatment with targeted therapies upon relapse. That there was no difference in locoregional control between the AGA and the non-AGA groups may be a true effect, as the mutations included in the AGA group of this study may have no radiosensitizing effect. Nevertheless, there are several reasons why this study may not have detected a difference. First, the non-AGA group was retrospectively randomly selected and therefore may be prone to undetected biases. Nevertheless, importantly, when the two groups were analyzed, the main prognostic factors were not significantly different, suggesting this was a fair comparator group. Second, owing to the retrospective nature of the study, the timing of follow-up scans to detect recurrence was center-dependent, adding variability in documenting the time to recurrence. Third, all mutations were grouped to ensure there were sufficient numbers for statistical analysis in this uncommon subgroup. This was thought appropriate owing to our hypothesis that tumors with an AGA spend more time in cell division, and therefore may have a greater susceptibility to ionizing radiation. Nevertheless, there is evidence to suggest that there is variation in local control of brain metastases according to AGA type, after stereotactic radiosurgery.^9^ The bulk of the patients with an AGA (70%) in our study had an *EGFR* mutation so if there was a strong radiosensitizing effect, it is likely to have been detected. Nevertheless, given the relatively small numbers of patients found with AGAs, it would be difficult to analyze AGA subtypes separately.

The finding that LRC is not improved in the presence of an AGA in this study contradicts in vitro experimental data^9^ and previous small clinical case series.^14^^,^^19^ In contrast with cohorts from Japan (n = 56) and France (n = 78), there was no difference in relapse locoregional relapse rates between patients with and without AGA. This observation could be explained by the inclusion of resectable cases in these cohorts, or that the Japanese study used a different RT modality (proton therapy). In contrast, all patients in the presented cohort were unresectable and were treated with curative-intent intensity-modulated photon RT. Another notable difference lies in the stages of patients included across these cohorts. Specifically, the Japanese cohort comprised individuals diagnosed with stage I to II NSCLC, while the French cohort exclusively focused on stage III cases. In contrast, the presented cohort encompassed patients across stages I to III. The relatively low rate of concurrent chemotherapy (35%) delivered with RT in this cohort somewhat restricts the transferability of these findings to centers with high rates of concurrent treatment. Notably, locoregional relapse occurred within six months of chemo-RT less frequently in patients with an AGA (6% versus 27%) potentially hinting at differential temporal patterns of local failure after chemo-RT.

The identification of a potential radiosensitization effect in prior smaller studies among patients with an AGA could justify exploring dose de-escalation approaches in this patient subset, in terms of dose or dose levels. Particularly when distant disease control is the predominant pattern of failure, adopting less aggressive RT strategies may effectively manage locoregional disease while potentially reducing treatment-related toxicity. Nevertheless, the results of the current study contradict previous literature, as local control was not different in patients with and without AGA. Our findings suggest that adopting dose de-escalation RT regimes could potentially compromise local control.

This current study largely focuses on those AGAs for which licensed targeted therapies were available during the study period. Nevertheless, other aberrations may have clinical relevance. For example, a single-center observational study in patients with stage III NSCLC found that alterations relating to genes involved in DNA damage repair may have utility in predicting a poor response to RT.^20^ The investigators found that deleterious mutations in genes including *ATM*, *MSH*, *ATR*, *BRCA*, *ERCC,* and *RAD50,* identified through an institutional gene panel, were associated with improved local control after RT. Cancer cell DNA damage induced by RT has the potential to be more lethal if left unrepaired, and certain drugs can target the DNA repair pathway. The CONCORDE phase Ib platform study is investigating multiple DNA damage inhibitors in patients with stage IIB to III NSCLC undergoing curative-intent RT.^21^ The primary objective of this innovative study is to determine the recommended phase II trial dose for these inhibitors.

We did not show an association between treatment with durvalumab and improved local control, relapse-free survival, or OS in patients with an AGA. Our findings align with recent evidence^3^^,^^22^ that adjuvant immunotherapy is not efficacious in patients with an AGA. Given the risk of heightened TKI toxicity after checkpoint inhibitor therapy, there is no rationale for durvalumab after chemo-RT, as indicated by the European Society of Medical Oncology recommendations.^23^ By contrast, the first landmark study evaluating adjuvant TKI therapy in stage III NSCLC with an AGA was published in 2024. LAURA was a phase 3, randomized placebo-controlled trial evaluating adjuvant osimertinib until progression or death versus placebo among patients with NSCLC and an exon 19 or 21 *EGFR* mutation, who completed definitive chemo-RT (concurrent or sequential).^8^ The study met its primary endpoint of progression-free survival with an impressive HR of 0.16 (*p* < 0.001). An additional 24% of patients had further tumor shrinkage as their best response in the experimental arm, compared to the control arm (57% versus 33%). The frequency of patients developing new lesions, of which most were extrathoracic, was decreased from 68% to 22% with osimertinib during the follow-up period. Together these data indicate that adjuvant TKI likely improves both local and distant control.

In addition to the consolidation TKI, there is a rationale for combining TKIs with RT for additive or synergistic effects.^24^ Several groups in Asia are investigating the combination of an alternate third-generation EGFR-targeted TKI, aumolertinib, in NSCLC treated with chemo-RT.^25^ A phase II trial is ongoing for concurrent plus adjuvant aumolertinib, with neoadjuvant aumolertinib for patients with with lung V20 >28% (NCT04636593). Disappointingly, a North American trial (RTOG–1306, NCT01822496) that investigated the impact of neoadjuvant TKI therapy before chemo-RT in patients with *EGFR* mutations or *ALK* rearrangements, closed early owing to poor recruitment. The concept of neoadjuvant TKI treatment is appealing as it theoretically offers the potential to reduce tumor volume, thereby potentially decreasing RT-related toxicity, enhancing disease control, and lowering the risk of early micrometastatic spread. The overlapping pulmonary toxicities of EGFR-targeted TKIs with thoracic RT imply that careful toxicity assessment is essential for such studies.^26^^,^^27^ In the current landscape, allowing for better access and quality of molecular profiling, a trial investigating neoadjuvant TKIs may stand a better chance of meeting its enrolment targets. Future studies could focus on the addition of targeted therapy in the concurrent setting to improve RT outcomes for patients with stage I to III NSCLC with AGAs.^27^

Improved access to molecular testing of liquid or tissue biopsies will facilitate better phenotyping of the AGA-positive cohort in the future. The frequency and characteristics of stage I to III disease with an AGA have not been established, but the implementation of molecular testing for non-metastatic cases in relation to ADAURA and LAURA highlights the importance of setting up systematic molecular testing in patients with localized NSCLC treated with curative intent. In centers with no access to liquid biopsies, the quality of biopsy samples will need to be maximized. The increased uptake of systematic staging should lead to greater yields of tumor content available for these tests.^28^ Molecular testing is required to make decisions about the indication for postoperative TKI and to decide whether a neoadjuvant treatment with chemo-immunotherapy is appropriate. The disappointing outcomes of immunotherapy in patients with an AGA means that multidisciplinary decision-making has become crucial in NSCLC, as the merits of resection versus chemo-RT plus adjuvant TKI have not been considered in head-to-head comparisons, with disease control, quality of life, and survival endpoints. Nevertheless, historical surgery versus RT trials have been associated with major recruitment challenges.^29^ Furthermore, conducting ADAURA- and LAURA-type studies in the less common AGAs is unlikely to be feasible given the small numbers of patients affected, combined with the high costs of running such trials. One option may be to promote umbrella study designs, which are more efficient, as exemplified by the NAUTIKA trial for neoadjuvant TKIs in AGA-positive NSCLC (NCT04302025). Extrapolation of the principle reported in *EGFR*-mutant disease to other AGAs, together with real-world evidence and preclinical signals, may be a pragmatic option for commissioning these treatment paradigms.^30^

Although there are many studies on the impact of radiotherapy for patients with metastatic disease, research on its role in localized disease remains scarce, primarily because molecular testing has not been routinely conducted in this setting. This study draws from the experience of four large cancer centers where reflex molecular testing for all stages of the disease was adopted early, providing a valuable perspective on an emerging field of radiation treatment for the non-metastatic mutation-positive disease. The strengths of this study lie in its multicenter design, the use of modern RT (99% intensity-modulated photon RT), the availability of detailed follow-up data, and the access to testing with broader panels beyond *EGFR* and *ALK*. Although less common AGAs are technically distinct, they share key characteristics with *EGFR* and *ALK* AGAs, such as early distant dissemination, sensitivity to targeted therapies, and associations with light smoking history. Furthermore, there is a lack of experimental data in this area to guide the stratification of the study population based solely on AGAs. In addition, this study represents, to our knowledge, the first evidence of the impact of AGAs on LRC within the immunotherapy era.

Beyond its retrospective nature, the main limitations are the insufficient power to distinguish AGA-specific patterns, the heterogeneity of AGA testing methods, and the lack of detailed RT treatment planning scan data, such as the spatial relationship between the high-dose region and areas of locoregional relapse. The low proportion of patients receiving chemotherapy as neoadjuvant or concurrent therapy, and durvalumab as adjuvant therapy in patients without an AGA, may restrict the transferability of these data to centers where these rates are higher. Unfortunately, no *NTRK*, *KEAP1*, or *NRG* alterations were identified. This was predictable, as during the study period, knowledge about these genes was limited, and it was not routinely included in testing panels. Furthermore, a detailed prospective registry study of relapse patterns may allow for personalization of follow-up according to AGA status. As such, these data should be interpreted with caution until a robust validation study is conducted.

## Conclusions

Patients with and without an AGA had comparable LRC rates in this study, suggesting that AGAs do not inherently enhance or diminish sensitivity to RT. As expected, patients with AGA had better OS, attributable to treatment with subsequent effective targeted therapies upon relapse. Our findings suggest that RT doses should not be tailored on the basis of AGA status. Further investigation is required to ascertain whether mutations in genes associated with DNA damage repair could provide insights into optimizing RT dose fractionation and synergistic drug combinations. As such these mutations should be considered for inclusion in future next-generation sequencing panels.

## CRediT Authorship Contribution Statement

**Salman Ashraf:** Data curation, Project administration, Validation, Writing - original draft, Writing - review & editing.

**Madeha Khan:** Data curation, Validation, Writing - review & editing.

**Nada Naguib:** Data curation, Validation, Writing - review & editing.

**Robert Rulach:** Data curation, Validation, Writing - review & editing.

**Katharine Welsh:** Data curation, Validation, Writing - review & editing.

**Rebecca Carozzi:** Data curation, Validation, Writing - review & editing.

**Ashley Horne:** Data curation, Validation, Writing - review & editing.

**Amelia Payne:** Data curation, Validation, Writing - review & editing.

**Sarah Bowen Jones:** Data curation, Validation, Writing - review & editing.

**Mary Denholm:** Investigation, Writing - review & editing.

**Georgia Stewart:** Investigation, Writing - review & editing.

**Ahmed Bedair:** Investigation, Writing - review & editing.

**Colin R. Lindsay:** Investigation, Writing - review & editing.

**Stephen Harrow:** Investigation, Writing - review & editing.

**Corinne Faivre-Finn:** Conceptualization, Formal analysis, Investigation, Methodology, Validation, Visualization, Project administration, Resources, Supervision, Writing - original draft, Writing - review & editing.

**Jonathan McAleese:** Conceptualization, Formal analysis, Methodology, Investigation, Writing - review & editing.

**Gerard G. Hanna:** Investigation, Writing - review & editing.

**Allan Hackshaw:** Conceptualization, Investigation, Methodology, Resources, Supervision, Writing - original draft, Writing - review & editing.

**Fiona McDonald:** Conceptualization, Investigation, Supervision, Writing - original draft, Writing - review & editing.

**Gerard M. Walls:** Conceptualization, Formal analysis, Investigation, Methodology, Validation, Visualization, Project administration, Resources, Supervision, Writing - original draft, Writing - review & editing.

## Disclosure

Prof. Hanna reports receiving a speaker honorarium from AstraZeneca. Dr. Walls reports receiving honoraria for conference analysis and speaker roles at non-promotional educational events for AstraZeneca. The remaining authors declare no conflict of interest.
